# Synthesis and Characterization of Polyaniline/Graphene Composite Nanofiber and Its Application as an Electrochemical DNA Biosensor for the Detection of *Mycobacterium tuberculosis*

**DOI:** 10.3390/s17122789

**Published:** 2017-12-02

**Authors:** Fatimah Syahidah Mohamad, Mohd Hazani Mat Zaid, Jaafar Abdullah, Ruzniza Mohd Zawawi, Hong Ngee Lim, Yusran Sulaiman, Norizah Abdul Rahman

**Affiliations:** Department of Chemistry, Faculty of Science, Universiti Putra Malaysia, 43400 UPM Serdang, Malaysia; fasya305@gmail.com (F.S.M.); zani.ukm@gmail.com (M.H.M.Z.); jafar@upm.edu.my (J.A.); ruzniza@upm.edu.my (R.M.Z.); hongngee@upm.edu.my (H.N.L.); yusran@upm.edu.my (Y.S.)

**Keywords:** *Mycobacterium tuberculosis*, polyaniline, graphene, nanofibers, biosensors

## Abstract

This article describes chemically modified polyaniline and graphene (PANI/GP) composite nanofibers prepared by self-assembly process using oxidative polymerization of aniline monomer and graphene in the presence of a solution containing poly(methyl vinyl ether-*alt*-maleic acid) (PMVEA). Characterization of the composite nanofibers was carried out by Fourier transform infrared (FTIR) and Raman spectroscopy, transmission electron microscopy (TEM) and scanning electron microscopy (SEM). SEM images revealed the size of the PANI nanofibers ranged from 90 to 360 nm in diameter and was greatly influenced by the proportion of PMVEA and graphene. The composite nanofibers with an immobilized DNA probe were used for the detection of *Mycobacterium tuberculosis* by using an electrochemical technique. A photochemical indicator, methylene blue (MB) was used to monitor the hybridization of target DNA by using differential pulse voltammetry (DPV) method. The detection range of DNA biosensor was obtained from of 10^−6^–10^−9^ M with the detection limit of 7.853 × 10^−7^ M under optimum conditions. The results show that the composite nanofibers have a great potential in a range of applications for DNA sensors.

## 1. Introduction

*Mycobacterium tuberculosis*, the etiological agent of tuberculosis (TB), is one of the infectious diseases that cause numerous deaths worldwide, with latent infection affecting as much as a quarter of the world’s population [1]. TB is a contagious airborne disease that can be spread from individual to individual through the air via exposure to coughs or sneezes [2]. In 2007, 13.7 million of serious cases were reported globally, with new infections spreading each year [3]. In addition, TB infection has become a chronic issue in some regions of the world, especially in developing countries, leading to an estimated 1.5 million deaths and 8.8 million new cases happened in 2010 [4]. The rate of TB is highest in many African countries, where an estimated 80% of the population test positive in tuberculin tests [5]. In Malaysia particularly, TB cases increased 6% in 2016 compared to 2015 with the total TB deaths jumping to 15% [6]. Thus, a quick, sensitive and rapid method for the detection of *Mycobacterium tuberculosis* would be a great of help to restrain the disease from spreading and quarantining patients.

Some of the common detection methods for TB infection include acid-fast staining [7], culturing on Lowenstein-Jensen media [8] and the real time polymerase chain reaction (PCR) [9]. However, these old conventional methods are not highly sensitive towards *Mycobacterium tuberculosis* pathogens and they also take a lot of time to be done due to the fact mycobacterial cultures usually require 4 to 8 weeks to achieve good growth [10]. Another analysis to diagnose TB is smear microscopy of sputum where the sputum fluid is taken from the lungs and along the esophagus [11]. The thick sputum fluid can be collected from patients through coughing. This method can provide results within hours, but the reported sensitivity is only about 50–60% [12]. In addition, chest X-rays, skin tests, fluorescent microscopy and serological tests [13] have also been used to detect TB infections, however, it takes a long time to get the results and the cost of examination is quite expensive due to the high maintenance of instruments. Consequently, biosensors have attracted great interest from researchers and their development plays an important role in daily life.

In recent years, a significant amount of research on biosensors based on *Mycobacterium tuberculosis* has been reported, including electrochemical sensors [14], piezoelectric biosensors [15], optical biosensors [16] and magneto-elastic biosensors [17]. Among the techniques, the electrochemical technique is relatively inexpensive and offers rapid detection [18]. Generally, electrochemical DNA biosensors are constructed by immobilization of single-strand DNA (ssDNA) probes onto an electrode to measure the hybridization between the ssDNA and their complementary target DNA. This step plays an important role in the performance of DNA biosensors. Modification of the electrode with nanomaterials is one of effective ways to further increase the sensitivity of biosensors [19]. Two types of probes which are DNA probes and peptide nucleic acid (PNA) probes can be employed to develop electrochemical biosensors [20]. Arora et al. have reported a method for the detection of *Mycobacterium tuberculosis* using PNA probes [21]. In this method, a 21-mer PNA probe specific to 16–23 s rRNA spacer region of *Mycobacterium tuberculosis*, has been covalently immobilized onto a polypyrrole-polyvinylsulphonate (PPy-PVS) film. The film was then electrochemically deposited onto indium-tin oxide (ITO) glass to form a PPy-PVS/ITO electrode. The PNA probe was used for the hybridization detection with complementary sequence of *M. tuberculosis* DNA with a detection limit of 2.5 pg/μL.

A number of studies have focused on polyaniline (PANI) conducting polymer nanostructures for catalytic materials and sensor development [22]. Among the unique properties of PANI are fast electron transfer dynamics and excellent electrochemical activity [23,24]. These unique properties allow PANI to serve as the direct electron mediator and further amplify the electrochemical signal. Meanwhile, graphene (GP) has the tremendously useful properties of a large surface area and excellent thermal conductivity [25,26]. GP can promote the transfer of electrons and further amplify electrochemical signals, which is very useful in sensor applications [27]. The unique properties of PANI together with the excellent properties of GP, motivated us to synthesis a PANI/GP composite nanofibers.

To the best of our knowledge, there are not many reports [14,28] that study conducting polymer nanofiber-graphene composite as a biosensor for the detection *Mycobacterium tuberculosis* and their properties as modified electrodes. This work reports the behaviour of polyaniline/graphene (PANI/GP) composite nanofibers as a DNA biosensor. The detection of *Mycobacterium tuberculosis* was based on the specific sequence of the DNA which has been confirmed as effective and sensitive [29]. Integrating PANI with GP composite nanofibers not only improved the immobilization of the DNA probe, but also enhanced the signal amplification. The properties of the modified electrode and its preliminary application as a DNA biosensor were also described in detail.

## 2. Materials and Methods

### 2.1. Reagents

The DNA oligonucleotides of *M. tuberculosis* were purchased as a lyophilized powder from First Base Laboratories Sdn. Bhd. (Shah Alam, Malaysia) and before use it should be reconstituted overnight at 2–8 °C to ensure that all material goes into solution. DNA probe and target stock solutions (100 µM) were prepared with Tris-EDTA (TE) solution (10 mM Tris-HCl, 1 mM EDTA, pH 8.00) and kept at −20 °C until used. Diluted solutions of DNA were prepared with 0.50 M phosphate buffer containing 20 mM NaCl (pH 7). The base sequences used are as below:

1. Probe DNA-single stranded DNA (ssDNA):
  5′-CTC gTC CAg CgC CgC TTC gg-3′
2. Complementary target DNA:
  5′-CCg AAg Cgg CgC Tgg ACg Ag-3′
3. Non-complementary DNA:
  5′-TTT GGT ATT ATT GTT CAT GT-3′.

Methylene blue (MB) was purchased from R&M Chemicals (Essex, UK) and the stock solution of MB (1 mM) was prepared in 0.1 M potassium chloride, KCl (pH 7.2); unless specific otherwise. More dilute solutions were prepared by using the same buffer solution. All other chemicals used were of analytical reagent grade. The supporting electrolyte used was 0.1 M KCl. Graphene powder and aniline were purchased from Aldrich (St.Louis, MO, USA). Activating reagents, 5 mM *N*-hydroxysulfosuccinimide sodium salt (NHS, Sigma Aldrich, Buchs, Switzerland) and 2 mM 1-ethyl-3(3′-dimethylaminopropyl)-carbodiimide (EDC, Fluka, Sydney, Australia) solution were prepared in 0.05 M phosphate buffer (pH 7.0). 5.0 mM potassium ferricyanide KFe(CN)_6_^3−^/^4−^ solution containing 0.1 M KCl (Aldrich, Dorset, UK) was used as the supporting electrolytes. 0.1 M phosphate buffer (pH 7.0) containing 20 mM NaCl (Aldrich, UK), 1 mM Na_2_HPO_4_ (Aldrich, UK), 2 mM KH_2_PO_4_ and 10 mM KCl (Aldrich, UK) were used as hybridization buffer. All chemicals were of analytical grade and used without further purification. All solutions were prepared with deionized water.

### 2.2. Synthesis of PANI and PANI/GP Nanofiber

The synthesis of PANI nanofibers was done according to a procedure previously reported by Zhang et al. [30]. A brief summary of the synthesis procedure is as follows: aniline (0.2 M) and PMVEA with different weight percentages of 0.5, 1, 3 and 6 wt % were dissolved in deionized water. Various concentrations of PMVEA were used in determining the effect of PMVEA concentration on the formation of PANI nanofibers. Then, the solution was cooled in a refrigerator at 4 °C for 60 min and then a pre-cooled aqueous solution of APS was added into the solution. The molar ratio of APS to aniline in the final solution was 1:1. PANI/GP nanofiber was synthesised using the same procedure except addition of GP powder into the solution to form nanocomposite. Weight ratios of GP to aniline were varied to 5, 10, 25 and 50%. The whole reaction for polymerization process took about 6 h to be completed when the precipitation of black-green PANI was observed. The products obtained were filtered and rinsed with methanol, deionized water and acetone at least three times. The final product was dried in vacuum overnight at a temperature of 40 °C.

### 2.3. Fabrication of DNA Biosensor

Before utilization, a screen printed carbon electrode (SPCE), was pretreated by using cycle voltammetry (CV) between 2 and −2 V for 40 cycles, scan rate of 100 mV s^−1^, in a 0.1 M KCl solution. Subsequently, PANI/GP nanocomposite solution (5 g/mL) was prepared by dispersing of PANI/GP powder in chloroform. 10 µl of the nanocomposite solution was drop casted onto the surface of SPCE and dried for 3 h at room temperature. Later, CV was performed using potential ranges between −1.0 V and 0.8 V for 10 cycles at scan rate of 100 mV/s. Subsequently, the modified SPCE/PANI/GP was dipped into a solution containing 5 mM of N-hydroxysuccinimide, (NHS) and 2 mM of 1-ethyl-3-(-3-dimethylaminopropyl) carbodiimide (EDC) for 1 h at room temperature. Then, 3 µL of DNA probe (10 µM) was dropped onto the surface of modified SPCE and incubated for 60 min at room temperature. Before conducting analysis by CV or differential pulse voltammetry (DPV), the SPCE surface was rinsed using phosphate buffer solution (PBS) containing 20 mM of NaCl (pH 7).

**Figure 1:**
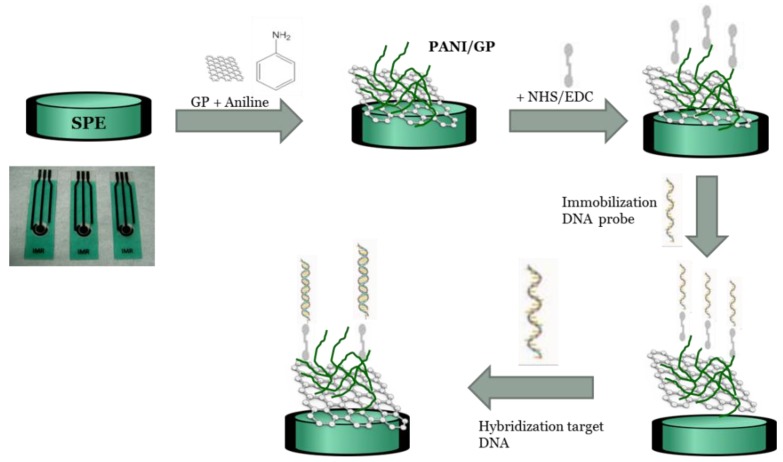
Schematic illustration of the stepwise electrochemical fabrication process for DNA biosensor.

Various concentrations of target DNA solution were dropped onto the surface of modified electrode and incubated for 45 min at room temperature. Then, the complete hybridized electrode was rinsed and washed using PBS (pH 7). Later, the hybridized electrode was immersed into 35 µM methylene blue (MB) in PBS (pH 7) with applied potential at +0.5 V for 10 min. After that, the electrode was rinsed with PBS to remove any unbound MB molecules. Figure 1 shows the fabrication process for the PANI/graphene TB sensor and the corresponding signal amplification of PANI/GP based on the DNA hybridization assays.

### 2.4. Electrochemical Measurements

All the CV and DPV measurements have been done by using a PGSTAT30 system (Metrohm Autolab, Utrecht, The Netherlands). The potential range used while performing CV was from −1.0 V to 0.8 V for 10 cycles at scan rate of 100 mV/s in KFe(CN)_6_^3−/4−^ solution containing 0.1 M KCl. Meanwhile, DPV was performed at potential range form −0.2 V to +0.8 V in 0.1 M PBS consisting 35 µM MB at pH 7.0. SPCE used in this research was bought from Rapid Lab Sdn. Bhd. (Putrajaya, Malaysia). Before carrying out CV and DPV the cell was purged with nitrogen gas for 10 min to allow the flow of nitrogen and block the oxygen from flowing back into the cell for the rest of the experiment.

### 2.5. Characterization

The morphologies of the products were analyzed with a JSM-7900 scanning electron microscope (JEOL, Tokyo, Japan). The samples for SEM were mounted on aluminium studs using adhesive graphite tape and sputter-coated with gold before analysis. TEM characterization was studied on a JEOL JSM-2100 instrument. The samples for TEM images were prepared by sonicating the samples in chloroform until equally distributed then the samples were drop casted on a copper grid covered with carbon. Infrared spectra were measured in the range 400–4000 cm^−1^ on PANI pellets made with KBr at a 1600 FTIR spectrophotometer (Perkin Elmer, Shelton, CT, USA) taking 10 scans at a resolution of 4 cm^−1^. Raman spectra were recorded using a RM 1000 laser Raman (He–Ne ion, Renishaw, West Dundee, IL, USA) containing a metallurgical microscope (Olympus Valley, PA, USA) and a CCD detector. The laser power at the sample was kept below ~0.74 mW to avoid thermal degradation.

## 3. Results and Discussion

**Figure 2:**
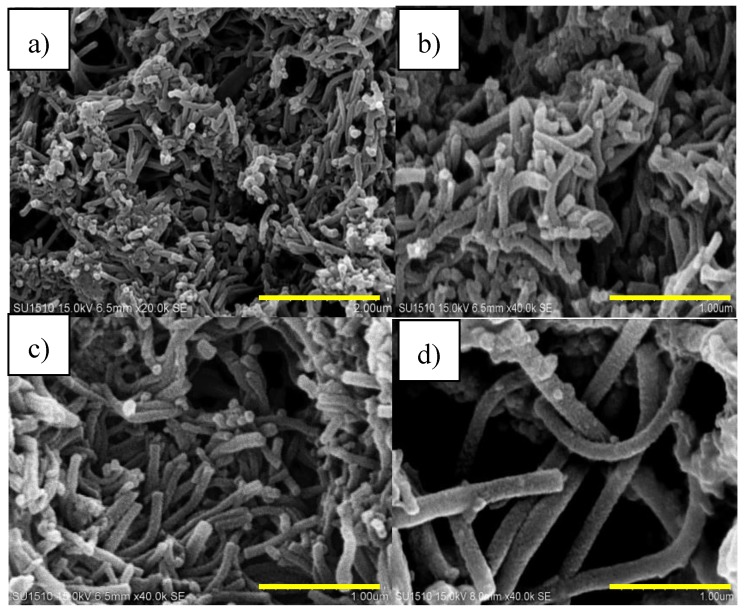
SEM images of PANI nanofibers at different wt% of PMVEA to aniline (**a**) 0.5 wt %; (**b**) 1 wt %; (**c**) 3 wt % and (**d**) 6 wt %.

Figure 2a–d show scanning electron microscope (SEM) images of the PANI nanofibers prepared from solutions that contained different weight fractions of PMVEA to aniline. The average fiber diameters of the nanofibers measured from the SEM images were 95 ± 10 nm, 170 ± 7 nm, 125 ± 12 nm and 360 ± 25 nm for 0.5 wt %, 1 wt %, 3 wt % and 6 wt % of PMVEA were used, respectively. The nanofibers diameter was increased with the increased of PMVEA. Several mechanisms have been reported for the formation of PANI nanofibers [31,32,33]. In this study, the formation of PANI nanofibers is a self-assembly process. The presence of acidic medium in the initial stages of reaction forms micelles or a template between aniline and acid [34,35]. Due to its surfactant properties, the polymeric acid PMVEA has the ability to stabilize the PANI dispersion [36,37]. Hence, it is reasonable to expect that PMVEA-aniline micelles may form and serve as nucleation sites for the growth of nanofibers.

The nanofiber formation starts from the micelle that acts as a ‘seed’ for nanofiber growth by accretion or an elongation process as previously suggested by Zhang and Wan [36]. Since the oxidant used in the polymerization (APS) is hydrophilic, the initial polymerization most probably occurred at the surface of water due to the solubility of APS in water. The number and diameter of the micelles would be depended on the concentration of the PMVEA surfactant as increasing the concentration of PMVEA will increase the size of micelles. Due to the small fiber diameter and uniform fibrous structure, PANI (3 wt %) was used in this study for incorporation with GP.

**Figure 3:**
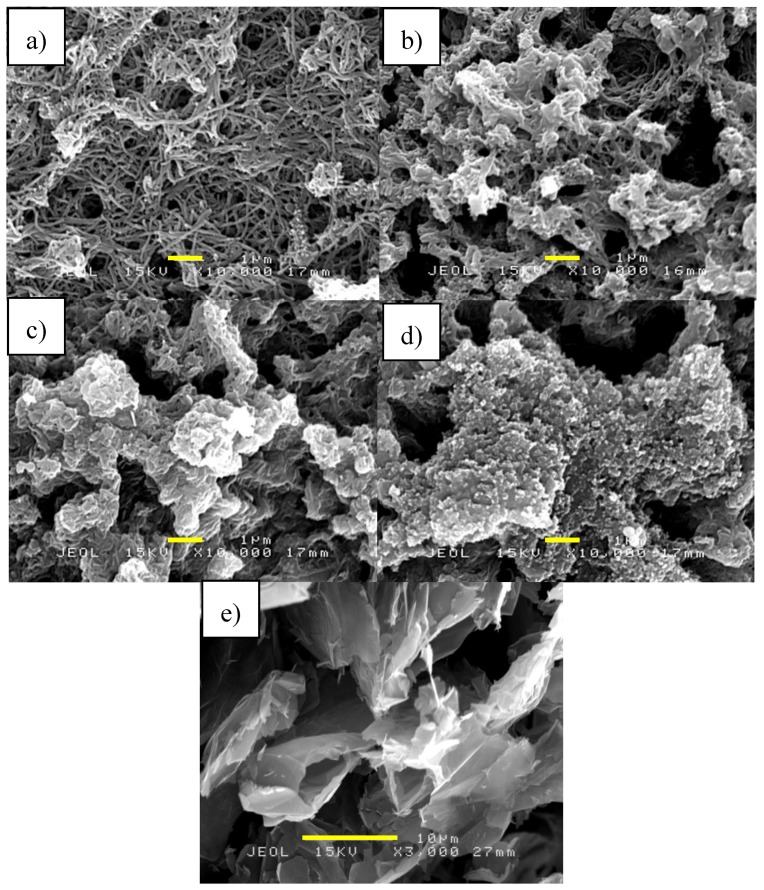
SEM images of PANI/GP nanofibers at different wt % of GP to aniline (**a**) 5 wt %; (**b**) 10 wt %; (**c**) 25 wt %; (**d**) 50 wt % and (**e**) graphene.

Figure 3 shows SEM images of GP and PANI/GP nanofibers at different GP contents. The SEM images of PANI/GP nanofibers shows uniform nanofibrous structures of PANI but agglomerated compared to PANI nanofibers alone. The SEM image of GP (Figure 3e) shows a good lamellar structure and rich wrinkled structures on the surface. At 5 wt % of GP, the SEM image of PANI/GP nanofibers show that GP sheets are homogeneously covered with PANI nanofibers. The PANI nanofibers distribute on the surface and between the GP sheets. As the GP composition increased to 50 wt %, the agglomeration of PANI nanofibers became bigger with less visible individual PANI nanofibers and PANI formed cauliflower-like structures that covered the GP. It indicates that the present of high composition of GP promote the formation of agglomerate PANI nanofiber. The SEM images also revealed that when the GP ratio increases, the nanocomposites also show an increasing crystalline structure, indicating that higher conductivity could be achieved in the composites consisting of higher GP loadings [38].

**Figure 4:**
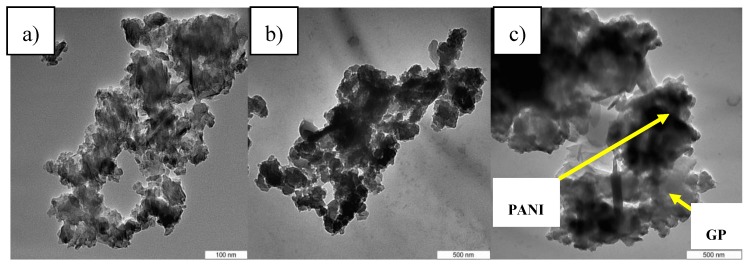
TEM images of (**a**) PANI nanofibers 3 wt % of PMVEA; (**b**) graphene and (**c**) PANI/GP 50 wt %.

Figure 4 shows TEM images of PANI nanofiber with 3 wt % of PMVEA, GP and PANI/GP nanofibers 50 wt % of GP. The incorporation of GP onto PANI can be observed in Figure 4c, where the darkest colour of the fibre indicates the presence of PANI and the greyish colour refers to the presence of GP [39].

**Figure 5:**
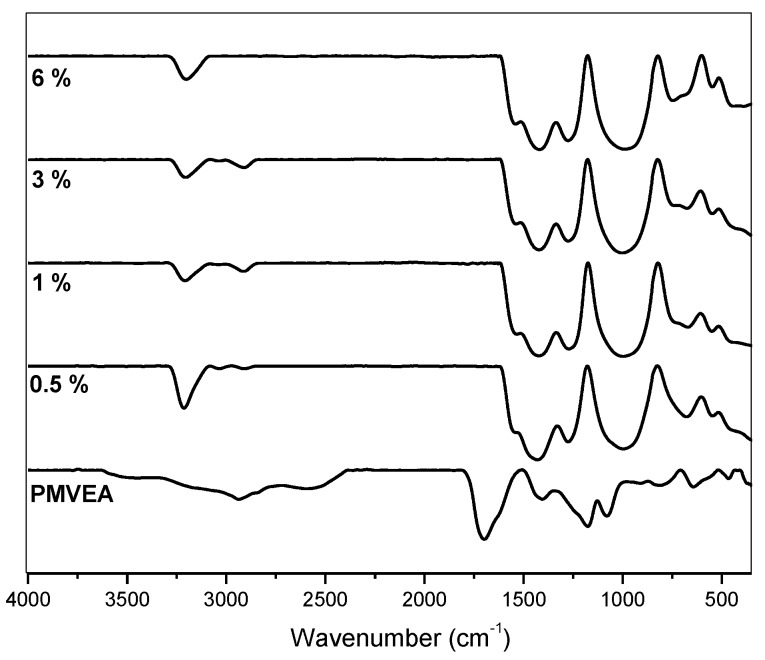
IR spectra of PMVEA and PANI nanofibers at different wt % of PMVEA.

Figure 5 presents IR spectra of PANI nanofibers obtained with varying contents of PMVEA and the IR spectrum of PMVEA for comparison. The FTIR spectra of PANI obtained from this research are similar to those previously reported [40]. The stretching deformation of C=C of quinoid and benzenoid rings are observed as bands at 1554 cm^−1^ and 1484 cm^−1^, respectively. A peak at 1310 cm^−1^ corresponds to the C─N stretching of the secondary aromatic amine. The band for C─H out-of-plane deformation in the 1,4-disubstituted benzene ring can be seen at 820 cm^−1^. Meanwhile, a band at 690 cm^−1^ belongs to the monosubstituted aromatic rings at the chain ends [41,42]. A characteristic band of the emeraldine salt form of PANI, which is C─N^+•^ stretching vibration in the polaron structure is observed at 1250 cm^−1^ [30,43]. A peak at 1700 cm^−1^ is commonly attributed to C=O stretching of the carboxyl group functionality found in the polymeric acid, PMVEA [44]. However, the peak disappeared in the IR spectra of PANI nanofibers indicating that PMVEA was washed out by the last step of PANI synthesis. This confirmed that PMVEA is no longer present in the PANI nanofibers.

**Figure 6:**
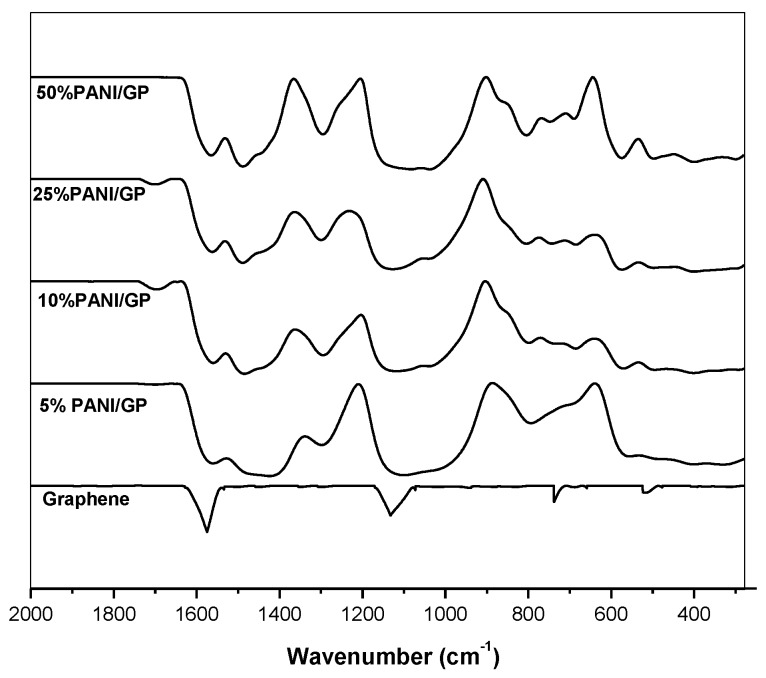
PANI/GP nanofiber at different wt % of GP to aniline.

Figure 6 shows the IR spectra of PANI/GP at various wt % of GP to aniline. The peaks at 1578 cm^−1^ and 1135 cm ^−1^ show the C=C and C─C stretches in GP, respectively. Compared to the IR spectra of PANI (Figure 4), the FTIR spectrum of PANI/GP in Figure 5 shows similarities to the IR spectra of PANI, probably due to overlap with the broad peaks of PANI. Peaks at 1290 cm^−1^, 1240 cm^−1^ and 1128 cm^−1^ indicate the stretching of the C─N in secondary aromatic amine, stretching of C─N^+^ and =N^+^─H, confirming that the PANI in PANI/GP is in a conducting form.

**Figure 7:**
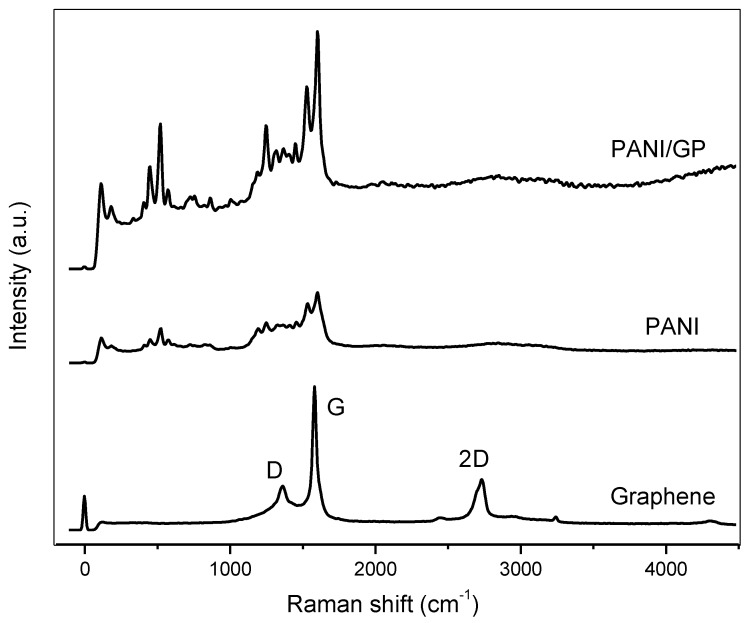
Raman spectra of GP, PANI nanofibers and PANI/GP.

Figure 7 shows the Raman spectra of GP, PANI and PANI/GP. The spectra show that the D band hybrid peaks (~1400 cm^−1^) and the G band (~1580 cm^−1^) of GP were merged together, and the defective signal of the D band increased notably while the intensity of the 2D band decreased compared with those of pristine GP. This could happen due to the coupling between PANI and GP through strong long-range π–π and electrostatic interactions [45]. The conversion of a sp^2^-hybridized carbon to a sp^3^-hybridized carbon is represented by D band and the in-plane bond-stretching motion of the pairs of C sp^2^ atoms is related to the G band.

**Figure 8:**
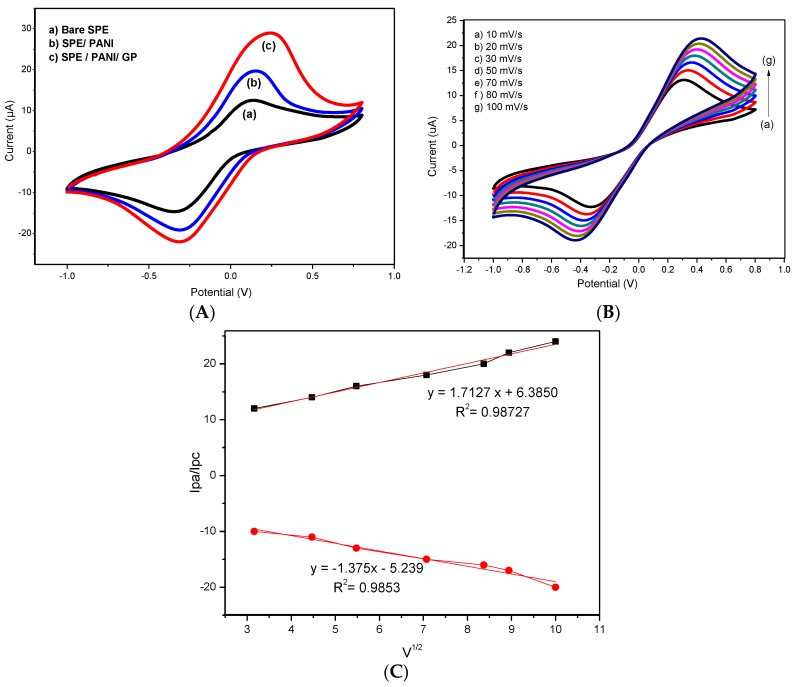
(**A**) Cyclic voltammograms of (**a**) bare SPCE (**b**) SPCE/PANI (**c**) SPCE/PANI/GP recorded in 5.0 mM Fe(CN)_6_^3−/4−^ solution containing 0.1 M KCl at 100 mV·s^−1^; (**B**) Cyclic voltammograms of PANI/GP at different scan rates (10–100 mV/s) in 5.0 mM Fe(CN)_6_^3−/4−^ and 0.1 M KCl at 100 mV·s^−1^ and (**C**) Plot of anodic and cathodic peak current (Ip) vs. square root of scan rate (V^1/2^).

In sensor applications, electrochemical characterization on the modified electrode plays an important role to evaluate the ability of the composite material in transferring electrons and enhancing the surface area. Therefore, it will affect the detection sensitivity of system used in this study. In this study, PANI/GP 50 wt % was chosen for the DNA sensor due to its high electrochemical properties due to the higher rate of redox reactions which gave the highest value of peak current compared to the other ratios (see Supplementary Data, Figure S1). Figure 8A shows the CV curves of bare SPCE, SPCE/PANI and SPCE/PANI/GP at scan rate of 100 mV/s, with the potential ranges of −1.0 to 0.8 V, in 5 mM potassium ferrocyanide ([Fe(CN)_6_]^3−/4−^)/1.0 M KCl. From the Figure 8A, we can see that an anodic and a cathodic peak were observed in all three electrodes. As shown in Figure 8A (curve c), the current response of SPCE/PANI/GP was higher than that of the SPCE/PANI (curve b) and bare SPCE (curve a). After modification of the SPCE electrode with PANI/GP composite nanofibers, the peak current increases, possibly due to the higher surface area and higher conductivities of both PANI and GP. Furthermore, the ability of diffusion control by Fe(CN)_6_^3−/4−^ ion on the electrode surface also affected the increment in peak currents [46]. The active surface area of modified electrode shows an improvement compared to bare electrode because of the higher acceleration electron transfer of Fe (CN)_6_^3−/4−^ ion on the roughness of the electrode surface due to the presence of PANI/GP [47].

CV of PANI/GP modified electrode was performed in 5.0 mM [Fe(CN)_6_]^3−/4−^ solution containing 0.1 M KCl at different scan rates of 10 to 100 mV/s in order to study the electroactive surface area (A) of electrode. Figure 8B,C show the plots of both anodic and cathodic currents from the plots of I vs. scan rate (V^1/2^). To calculate the electroactive surface area of the electrode, we used Randles–Sevcik equation [48].

Ip = 2.69 × 10^5^A × D^1/2^n^2/3^Cv^1/2^

where Ip represents the maximum current (in Amperes), n = number of electrons transferred (in this work n = 1), D = diffusion coefficient (cm^2^ s^−1^) of [Fe(CN)_6_]^3−/4−^ solution (7.6 × 10^−6^ cm^2^ s^−1^) [49], A = electrode area (cm^2^), C = concentration (mol cm^−3^) and v = scan rate (mV/s). The electroactive surface area (A) of the bare SPCE calculated to be at 0.0065 cm^2^, while electroactive surface area for SPCE/PANI/GP is 0.0124 cm^2^. The active surface area for the modified electrode consisting PANI/GP increased approximately two-fold compared to the bare electrode. In addition, straight lines form in both the anodic and cathodic currents for the plots of I vs. square root of scan rate (V^1/2^) as shown in Figure 8C, proving that a diffusional process has taken control in the reaction of ferrocyanide/ ferricyanide.

### Electrochemical Characterization of the DNA Biosensor

To further study probe coverage and surface modification, MB has been used as a redox label due to the affinity of MB towards DNA. Generally, the time used for the immersion of MB was studied in the range of 10 to 120 min [50]. In this study, the 60 min was chosen as the period for the immersion of MB. Several mechanisms have been studied for the interaction of MB, such as an interaction between guanine bases in the ssDNA [51,52] or by an electrostatic interaction with the negatively charged phosphate groups [53]. By using MB in electrochemical reaction can minimize the background and interferences from other electroactive species compared to the detection with label-free electrochemical method.

**Figure 9:**
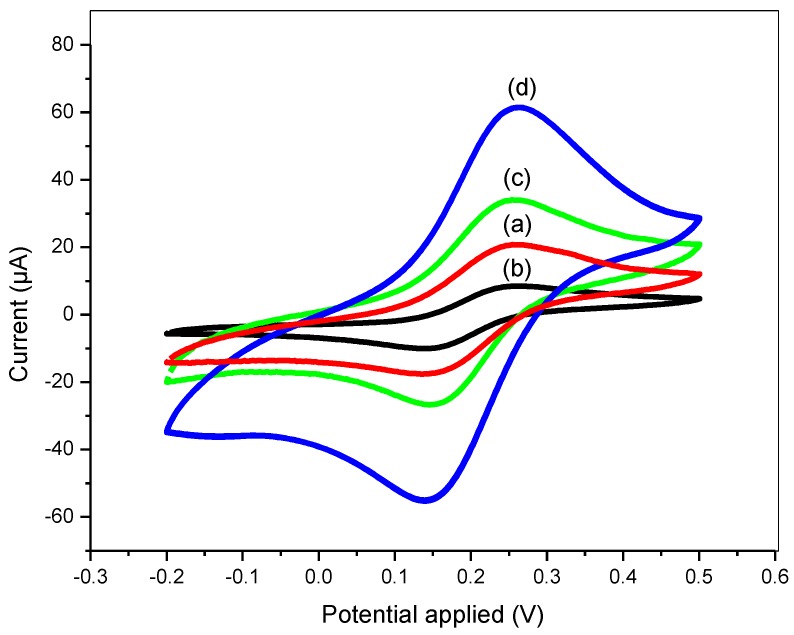
MB current peak of the DNA biosensor by CV (**a**) SPCE/PANI/GP (**b**) SPCE/PANI/GP/ EDC/NHS (**c**) SPCE/PANI/GP/EDC/NHS/DNA probe (**d**) SPCE/PANI/GP/EDC/NHS/DNA-DNA target with 35 µM of MB at potential −0.2 V to 0.5 V using scan rate of 100 mV/s in 0.1 M PBS buffer.

Figure 9 shows the CV of SPCE/PANI/GP, SPCE/PANI/GP/NHS/EDC, SPCE/PANI/GP/EDC/ NHS/DNA probe and SPCE/PANI/GP/EDC/NHS/DNA probe-DNA target in the presence of MB. After treating the modified electrode with 5 mM NHS and 2 mM EDC, a decrease in peak current was observed (curve b), due to its non-electroactive properties blocking electron transfer by forming carbodiimide coupling linkers. However, in curve c an increase of the peak current was observed according to the immobilization of thiolated ssDNA probe onto SPCE/PANI/GP modified electrode. When the target DNA solution was dropped onto the electrode surface for 15 min, the peak current further increased, proving that the oligonucleotides of the target DNA were successfully hybridized with the DNA probe (curve d).

Several mechanisms have been reported regarding the interaction between MB and ssDNA based on the electrochemical behavior [28,54]. Fundamentally, the most reported MB binding modes based on their binding to nucleic acid are electrostatic interactions between cationic MB and anionic DNA, intercalation of MB in the major or minor grooves of dsDNA helix and preferential binding between MB and guanine bases [55]. The increment or decrease of the MB signal after hybridization with target DNA is due to either vertical or horizontal orientation of the conformation DNA presents on the electrodes [56,57]. These kinds of orientation will determine the interaction of MB with the guanine before hybridization even after the formation of DNA duplex for flat probes such as gold electrodes [57]. In this work, a large increase of the oxidation peak current after hybridization with target DNA was observed, (Figure 9), which indicated the accomplishment of the hybridization reactions on the surface of the modified electrode. However, the orientation of the DNA duplex conformation on the electrodes cannot be determined and needs further study since our system consists of a 3D probe structure, which is different from a flat probe.

**Figure 10:**
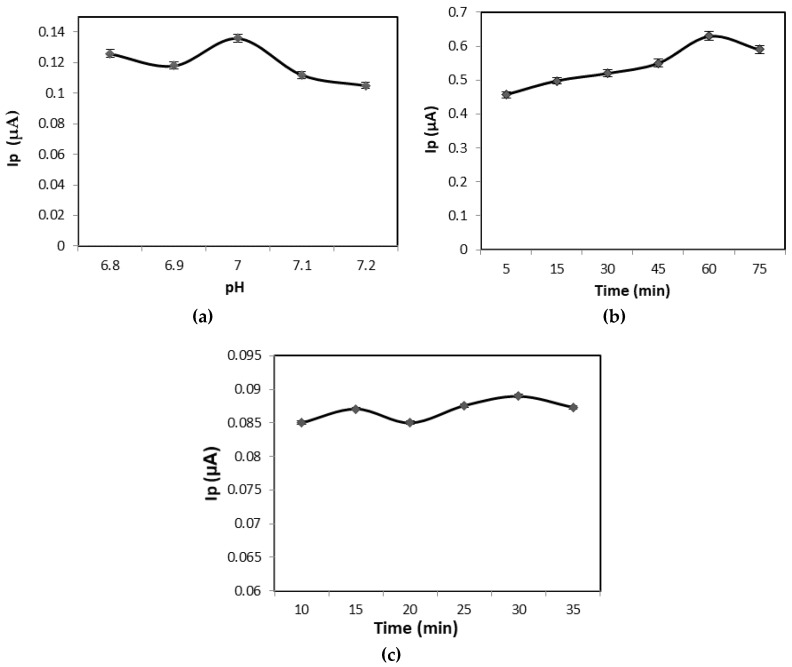
Differential pulse voltammograms (DPV) of DNA biosensor during optimization parameters (**a**) pH of buffer solution; (**b**) immobilization time of DNA probe, and (**c**) hybridization time of DNA target in phosphate buffer (0.1 M, pH 7) consisting MB (35 µM) at potential −0.2 V to 0.8 V.

In this study, the performance of the developed biosensor was further investigated by optimization of several parameters including the immobilization time of probe DNA, hybridization time with target DNA and pH of the working buffer solution. As shown in Figure 10a, the voltammetric signal of SPCE/PANI/GP/EDC/NHS/DNA probe showed a pH dependent bell shape. The cathodic current reduced slightly from 0.126 µA to 0.118 µA from pH 6.8 to 6.9. Then, there was an increase to the optimum current of 0.136 µA as the pH of 1 M PBS increased from 6.9 to 7.2. After that, the current started to reduce again as the pH increased to 7.2. Hence, the neutral pH 7.0 has been chosen as the optimum pH for hybridization of DNA probe with target DNA.

Figure 10b,c display the DPV cathodic current of the SPCE/PANI/GP/EDC/NHS/DNA probe at various sDNA probe immobilization times and target DNA hybridization times, respectively. As shown in Figure 10b, as the immobilization time increased, the cathodic peak current also increased. It was found that 60 min is the optimum time for DNA probe immobilization. Beyond 60 min, the peak current reached its saturation point, where the current starts to decrease. The effect of hybridization time was also studied in the range of 10–35 min. Figure 10c shows that the oxidation current fluctuates until it reaches the optimum current peak at 30 min of hybridization. Considering the hybridization efficiency, 30 min was selected as the optimum time for the hybridization process of the DNA target onto the modified electrode.

**Figure 11:**
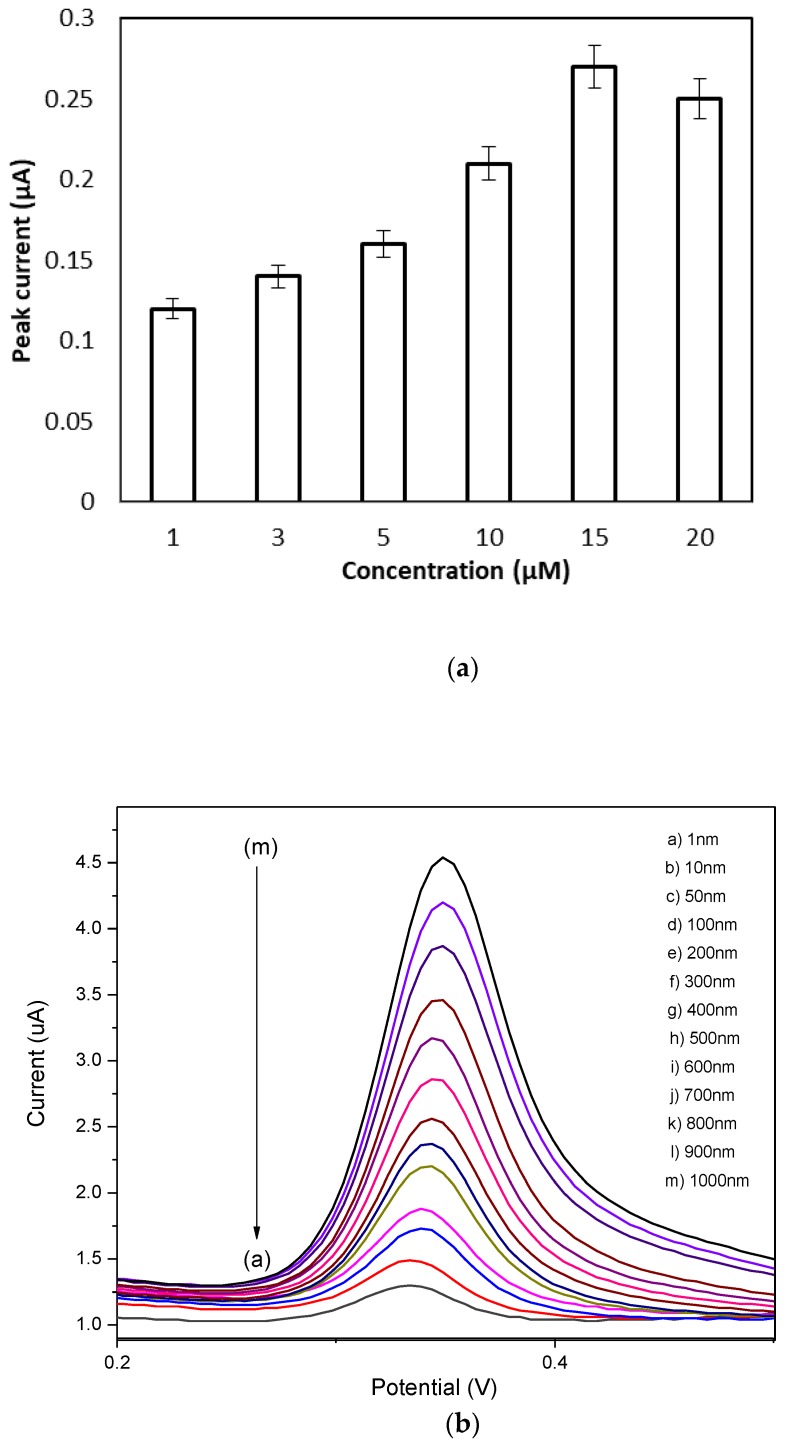

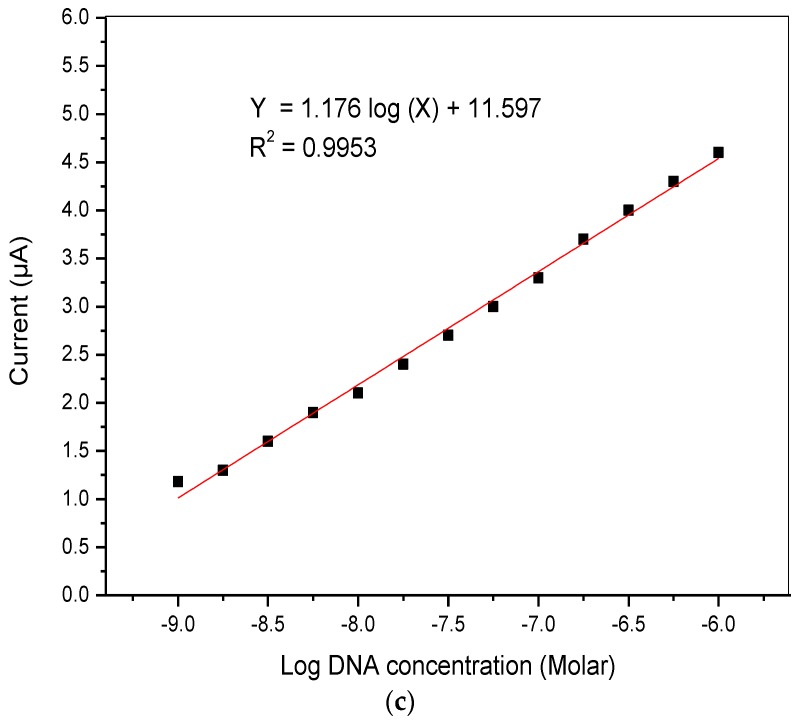
(**a**) Histogram for DPV measurement of different concentration of DNA probe (1–15 µM); (**b**) DPV measurements of different concentration of DNA target (from a to m were 10^−6^ M–10^−9^ M) in phosphate buffer (0.1 M, pH 7) consisting MB (35 µM) at potential range (−0.2 V to +0.8 V); (**c**) plot of anodic peak current against log concentration of DNA target.

Figure 11a shows DPV response of SPE/PANI/GP/EDC/NHS at various concentrations of DNA probe 1 µM to 20 µM at fixed target DNA (10^−6^ M). Meanwhile, Figure 11b shows a DPV response hybridization of SPE/PANI/GP/EDC/NHS/DNA probe at different concentrations of DNA target from 10^−9^ M to 10^−6^ M at a constant DNA probe concentration of 15 µM. All the reactions during immobilization and hybridization of DNA were done in phosphate buffer (0.1 M, pH 7) containing MB (35 µM). Both of the Figure 11a,b show an increase in peak current as the concentration of DNA probe and DNA target was increased. As shown in Figure 11a, the voltammetric signal increased from 1 µM to 15 µM of probe DNA. As the probe concentration increases, more probes were immobilized onto the electrode, and the maximum oxidation current was obtained at 15 µM, so the probe concentration was chosen as 15 µM as it was the optimum concentration for DNA probe to completely accumulate onto the SPE surface.

As shown in Figure 11b, when the concentration of complementary target DNA is increased from 10^−9^ M to 10^−6^ M, a considerably increased peak current was observed from 1.25 µA to 4.57 µA, respectively. Meanwhile, in Figure 11c, the plot of the target DNA concentration relationship can be written as Y = 1.176∙log(X) + 11.597 with a correlation coefficient of R^2^ = 0.9953, where Y is the peak current and log X is the logarithm of the target DNA concentration. The detection limit was found to be at 7.853 × 10^−7^ M, which was calculated based on 3 σ divided by slope, where σ is the standard deviation of the blank (n = 3) [58].

**Figure 12:**
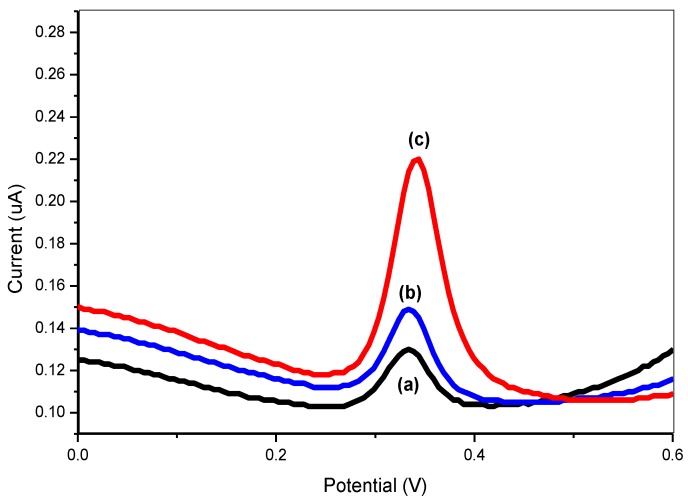
The DPV of (**a**) PANI/GP-DNA probe in non-complementary DNA; (**b**) PANI/GP-DNA probe, and (**c**) PANI/GP-DNA probe-complementary DNA in 0.1 M buffer solution and consisting MB (35 µM).

To investigate the selectivity of the designed biosensor towards the target DNA, the DNA probe was hybridized with complementary target DNA and non-complementary DNA. As illustrated in Figure 12, the MB signal shows a relatively small current when non-complementary DNA was introduced, indicating that non-specific hybridization between DNA probe and non-complementary target had occurred. 

However, an obvious increase of the MB oxidation peak current was observed when DNA probe was hybridized with complementary target DNA, which clearly shows the successful complementary binding between DNA probe and DNA target. This demonstrated that the designed DNA biosensor could recognize and detect the target *Mycobacterium tuberculosis* DNA.

In this work, the stability of the DNA biosensor was investigated by repetitive recordings of CV measurements in PBS after incubation with 100 nM target DNA at potential range −0.2 V to 0.8 V for 50 cycles. The RSD (%) was determined as 4.76 and 4.99% showing that the modified electrode is reusable and can be used several times. Additionally, we demonstrated the stability of the biosensor using a long-term storage assay. The electrochemical signal retained 91.7% of its initial current after 30 days of storage at 4 °C, indicating that the stability was sufficient for use in *Mycobacterium tuberculosis* detection. Moreover, to study the reproducibility of the fabricated biosensor, six modified electrodes (SPE/PANI/GP/EDC/NHS/DNA probe) were fabricated independently with the same procedure and hybridized with 10^−6^ M complementary target DNA. RSD of the oxidation peak current was calculated to be at 2.5% (n = 5), showing the excellent reproducibility of the fabricated DNA biosensor.

## 4. Conclusions

This research study describes a simple, fast and sensitive procedure using PANI/GP composite nanofiber as a biosensor for the detection of *M. tuberculosis* DNA. In this work, we demonstrated an electrochemical detection method based on the hybridization of DNA probe with the complementary DNA target of *Mycobacterium tuberculosis* using DPV. From the results, the limit of detection for hybridization of ssDNA probe and ssDNA target (10^−6^ M to 10^−9^ M) is 7.853 × 10^−7^ M. The results showed a good electrochemical activity of PANI/GP composite nanofibers due to their high sensitivity and specificity with synthetic DNA. The proposed DNA biosensor has shown high potential for the detection of *Mycobacterium tuberculosis*.

## Supplementary Materials

The following are available online at http://www.mdpi.com/1424-8220/17/12/2789/s1. Figure S1: CV of PANI/GP with different weight ratios at scan rates 100 mV/s in 5.0 mM Fe(CN)_6_^3−/4−^ and 0.1 M KCl at potential −1.0 V to 0.8 V.
