# Supplementary material for: Synthesis and Characterization of Polyaniline/Graphene Composite Nanofiber and Its Application as an Electrochemical DNA Biosensor for the Detection of Mycobacterium tuberculosis
Source: Sensors (Basel). 2017 Dec 2;17(12):2789. doi: 10.3390/s17122789 (PMC5751647; doi:10.3390/s17122789)
Supplement: Supplementary file 1 [file sensors-17-02789-s001.pdf]

Supplementary Data

Synthesis and Characterization of Polyaniline/Graphene Composite Nanofibers and Its Application as Electrochemical DNA Biosensor for Detection of Mycobacterium Tuberculosis.

Fatimah Syahidah Mohamad, Hazani Mat Daud, Jaafar Abdullah, Ruzniza Mohd Zawawi, Lim Hong Ngee, Yusran Sulaiman and Norizah Abdul Rahman*.

Department of Chemistry, Faculty of Science, Universiti Putra Malaysia, 43400 UPM Serdang, Selangor, Malaysia
*Correspondence: a_norizah@upm.edu.my

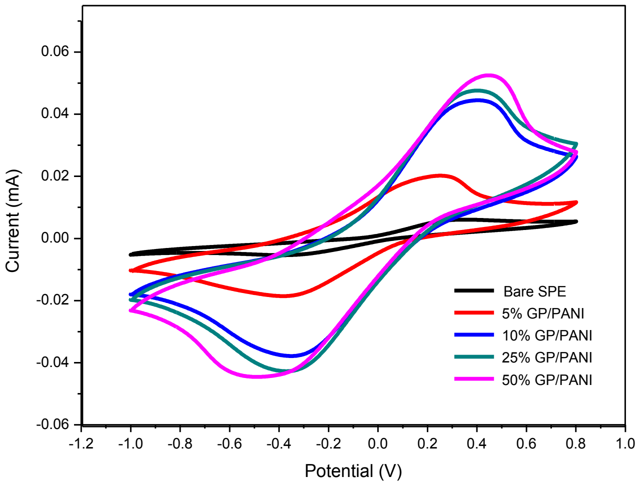

Figure S1: CV of PANI/GP with different weight ratios at scan rates 100 mV/s in 5.0 mM $Fe(CN)_6^{3-/4-}$ and 0.1 M KCl at potential -1.0 V to 0.8 V.
